# Gambling, housing conditions, community contexts and child health in remote indigenous communities in the Northern Territory, Australia

**DOI:** 10.1186/1471-2458-12-377

**Published:** 2012-05-25

**Authors:** Matthew Stevens, Ross Bailie

**Affiliations:** 1 Menzies School of Health Research, Institute of Advanced Studies, Charles Darwin University, Darwin, Australia

**Keywords:** Gambling, Child health, Indigenous, Public health

## Abstract

****Background**:**

Recent government reports have identified gambling, along with alcohol abuse, drug abuse and pornography, as contributing to child neglect and abuse in Indigenous communities in the Northern Territory (NT). These reports also identify gaps in empirical evidence upon which to base sound policy. To address this shortfall, data from ten remote Indigenous communities was analysed to determine the relationship between gambling problems, housing conditions, community contexts and child health in indigenous communities.

****Methods**:**

Logistic regression was used to assess associations between gambling problems, community contexts, housing conditions and child health. Separate multivariable models were developed for carer reported gambling problems in houses and six child health outcomes.

****Results**:**

Carer reported gambling problems in households across the ten communities ranged from 10% to 74%. Inland tropical communities had the highest level of reported gambling problems. Less access to a doctor in the community showed evidence of a multivariable adjusted association with gambling problems in houses. No housing variables showed evidence for a multivariable association with reported gambling problems. There was evidence for gambling problems having a multivariable adjusted association with carer report of scabies and ear infection in children.

****Conclusions**:**

The analyses provide evidence that gambling is a significant problem in Indigenous communities and that gambling problems in households is related to poor child health outcomes. A comprehensive (prevention, treatment, regulation and education) public health approach to harm minimisation associated with gambling amongst the Indigenous population is required that builds on current normative community regulation of gambling.

## **Background**

Card games have been an ubiquitous activity in remote Aboriginal communities across the Northern Territory (NT) for over sixty years [1]. Gambling, as an activity does not sit outside the social and physical environments that it is played. Both *how* games are played and the *consequences* arising can be affected by the environment in which it is played [2-5]. While researchers have acknowledged the interactions between social and physical environments and gambling, few studies have incorporated research designs that measure and control for the differing environments in which gambling exists. This paper explores the complex associations between gambling problems, child health outcomes, housing conditions and community contexts using a rich data set from ten Indigenous communities in the NT.

### **Consequences of community card games**

Research in the NT up until the early 1980s found gambling to be a relatively benign activity that allowed Aboriginal people to express traditional kinship ties of sharing and was perceived as a good fit with hunter and gatherer belief systems-namely luck [2,6]. Card games were generally played for fun as a social activity, particularly at family gatherings, though higher stake games were played as a way of accumulating sums of money for more costly purchases such as white goods [2]. However, studies in remote communities in north Queensland and Western Australia highlighted a variety of negative consequences associated with card games [4,7-9]. In particular, the boom and bust cycle associated with losing and then having a big win, increased family tensions and an erosion of traditional kinship relations, and children not attending school were noted in these studies [4,8].

Positive and negative consequences arising from community gambling do not occur in isolation from broader community social and physical structures. Breen et al. [3] conducted research in north Queensland Indigenous communities, and found considerable normative regulation of card games. This included separation of high and low stake games, duration and frequency of games, location of games and type of game played (e.g. fast paced versus low paced, and chance versus skill). Card games tend to be more frequent and higher stake games when there was an influx of money into the community through either regular social security payments or through irregular payments such as tax returns, royalties and the sale of art works. As such, there tends to be a fortnightly cycle of gambling, peaking on pay days and petering off over the following days after which games tend to be lower stake and longer duration. In addition to the normative regulation, Breen also classified gamblers as committed, exploited, binge and social. These categories of gambler were spread across a continuum of unhealthy gambling (committed), through to healthy gambling (social).

The social element of card games was evident in the recent north Queensland study, where gatherings for card games still served an important role for story-telling, yarning and catching up on gossip [3]. These social games were not considered to cause significant harm and were viewed as important places where people could discuss community issues, in addition to story-telling. However, the games in which ‘committed’ and ‘exploited’ gamblers participated were seen to cause significant harm for participants. Specifically, the study found that exploited gamblers were usually elderly or people on disability pensions, and were often coerced to play in games of skill with skilful gamblers, where they inevitably lost [3].

A difficulty with reaching firm conclusions on the negative and positive aspects of community card games results from differing research methodologies being used, and very few larger-scale empirically based studies linking card gambling to poor psychosocial or health outcomes. The Australia Bureau of Statistics (ABS) collects some data on gambling problems as part of its social survey program. Respondents are asked if ‘gambling problems’ have been a problem for themselves, a family member or a close friend in the last year. Across Australia in 2002, 26% of Indigenous people in remote localities reported gambling problems, while the same figure was 10% for the non-remote Indigenous population, compared with 3.5% within the general population [10,11]. In the NT, the same figures for remote Indigenous, non-remote Indigenous and the general population were 32%, 11% and 3% respectively [12]. While the percentage of the Indigenous population reporting gambling problems is much higher than in the general population in these surveys, there were many similarities in the significant predictors of gambling problems. For example, being a victim of actual or threatened violence, and participation in social activities were associated with gambling problems for both the Indigenous and general populations for Australia and the NT [12,13].

### **Child health and community gambling**

The enmeshed nature of gambling has distinct implications for remote Indigenous communities where the broader community environment is known to affect people’s health and emotional and social wellbeing (ESWB) [14-17]. Given the social nature of gambling, there is potential for houses being used as card game venues in remote communities to become overcrowded, which will lead to more wear and tear on physical infrastructure in the house [17]. Therefore, indirectly, gambling has the potential to affect people’s ability to carry out healthy living practices and maintain the hygienic condition of houses which will have health implications for the occupants of the house, and particularly so for children [15,16,18-20]. Further, gambling houses are more likely to have a steady flow of people coming through the house, leading to increased possibility of transmission of infectious diseases either through direct contact or the sharing of linen and towels [21].

Community and household problems are often exacerbated by overcrowded living conditions, where one person’s problem is more likely to affect a larger number of people living in the same house. Hoy et al. [22] conducted a brief health survey of adults in a large remote Indigenous community in the 1990’s and found high rates of infectious diseases including skin sores (25%) and scabies (17%), ear discharge (approximately 25% and at least one ear drum perforated in 20%) and a loose cough (approximately 50%). The authors identified a range of factors contributing to these high rates including: crowding, poor hygiene, poor host defences (associated with nutrition) and high bacterial loads. Poor access to healthy foods were a problem for this community, but it was noted that ‘diversion of money to beer, gambling and cigarettes and poor food choice remain fundamental obstacles to good nutrition’ [22], p125. These problems, combined with high rates of infectious disease all elevate the health risks for children in these communities.

The *Little Children are Sacred* report recently highlighted high levels of child neglect in Indigenous communities in the NT and raised concerns about negative consequences arising from gambling [23]. The report highlighted the lack of good evidence on the relationship between gambling (problems) and child health to inform more immediate and direct policy responses. The recent review of child protection services in the NT again identified gambling as a factor contributing to child neglect in Indigenous communities [24]. The interaction between gambling and child health and neglect is best illustrated through the following quote from the summary report.

"*Environments where there is substance abuse and where gambling is prevalent will also impact on parental vigilance and the supervision of children, and can involve many strangers in the home, leading to an impact on children’s health and wellbeing by increasing children’s access to drugs, alcohol and drug paraphernalia.*[24]"

This quote highlights the interaction between gambling, the transmission of infectious diseases, and broader issues of child neglect that are too common in remote Indigenous communities in the NT. So, while there is anecdotal evidence of an association between gambling, child neglect and poor health, there is little empirical evidence to support this assertion (though see [4]).

The Housing Improvement and Child Health in Aboriginal communities study (HICH) is a rich data set containing information on community, carer, householder, dwelling and child characteristics for ten remote Aboriginal communities in the NT [15,17,25]. The HICH data set contains a similar question on reported gambling problems as collected in ABS surveys and will be utilised in this paper. Specifically, the analyses in this paper will (i) identify housing, carer and community contexts that show evidence of an association with carer reported gambling problems, (ii) identify community traits that show evidence of mediating carer report of gambling problems, and (iii) determine whether there is evidence of reported gambling problems having a multivariable adjusted association with poor child health. Results are discussed and strategies to minimise harm associated with gambling in Indigenous communities suggested.

## **Methods**

### **Study sites, design and questionnaires**

Ten Aboriginal communities were selected for a study of housing improvement and child health in 2004–5. Communities were spread across the NT, and included coastal, inland tropical and desert communities ranging in size from 250 to 1,450 people, with an average population of 730. Communities were selected because they were known to be receiving significant housing upgrades and the primary research question of the HICH study was whether improved housing was associated with an improvement in child health [15,17,18]. Therefore, the study represents prospective cohort design in which children were followed up; however, for the current analyses only baseline data are used and is therefore cross-sectional in nature. The scope of the study included all children less than 7 years of age. Consent for children in the study was obtained from their primary carer. Consent was also obtained separately from all carers' and householders' participating in the study.

The HICH study was designed to collect detailed information on housing conditions, and importantly on carers, householders, children and community contexts to allow for the complex relationships that exist to be accounted for in statistical analyses. The use of the HICH data to explore the complex relationships between carer characteristics, housing conditions, community factors, child health and reported gambling problems is consistent with the overall goals of the study in gaining a better understanding of how household and community environments affect child health in Indigenous communities in the NT.

Six survey instruments were used to collect data on the: 1) community, 2) householder (head of house), 3) dwelling, 4) primary carer of child, 5) child health and demographics (carer report), and 6) child health (audit of health clinic data). Carer, householder and child information were all collected through face-to-face interviews and in all communities local interpreters were employed to assist in translation of the survey questions when interviews were being carried out. The survey instruments were piloted in two communities prior to commencing the survey and modification and deletion of questions made as required [16].

Ethics approval (Application: 02/66) for the project was obtained from the Human Research Ethics Committee of the Northern Territory Department of Health and Menzies School of Health Research (Registration: EC0153) and the Central Australian (no reference number supplied) Ethics Committee, and formal agreements to participate were signed by peak organisations in each of the ten Indigenous communities.

### **Primary outcome variables**

*Child health* was measured by asking the primary carer of the child about the presence or absence of common childhood illnesses for the two weeks preceding the survey. From this, six child health variables were constructed and are: 1) skin infections with no scabies, 2) scabies (with or without skin infections), 3) respiratory infections, 4) diarrhoea and/or vomiting, 5) ear infections, and 6) a composite child health variable indicating that the carer reported two or more of the five illnesses for the child. The study also collected child health data through an audit of health centre data; however, exploratory data analysis revealed this data to be less reliable than carer report, due to differential recording of illness between community health centres (see [15,18]).

*Reported gambling problems* were measured using the Negative Life Events Scale (NLES), which was included in the carer and householder surveys. The NLES measures a person’s exposure to ‘life stressors’ and was designed for use in surveys of both the Indigenous and non-Indigenous populations of Australia. The scale was developed by the ABS in consultation with a special advisory group that guided items for inclusion. This group comprised experts in Indigenous information, research and cultural issues, who were nominated “from Indigenous organisations, peak Indigenous information bodies, Commonwealth and state/territory government agencies with Indigenous program responsibilities, and relevant academic research institutions” [26], p. 1. The NLES, as used in ABS surveys asks respondents *since last* [insert current month] *last year, have any of these* [list of stressors] *been a problem for you, or your family or friends?* However, for the HICH study this question was modified to include only the respondent and any one in the house in which they reside. Specifically respondents were asked *if any of these things* [list of stressors] *has been a problem for yourself or someone in this house in the last 12 months*. Respondents were then shown/read out a list of ‘negative life events’, or ‘life stressors’ for which a yes or no response was elicited. The stressors included: gambling problem; alcohol or drug related problems; being a witness to violence; being abused or in a violent crime; trouble with the police; divorce or separation; not able to get a job; lost a job or sacked; death of family member or close friend; serious illness or disability in a family member; serious accident by family member; overcrowding; racism or treated different; and vandalism.

The NLES was administered to both the carer and householder, so the reported gambling problems variable was available for the carer and householder. A community level gambling problems variable was also generated indicating that carer’s report of gambling problems in households for the community were: (i) less than 20%, (ii) 20–49%, and (iii) 50% or more. Both the carer and householder report of gambling problems yielded very similar estimates for communities, so only the carer reported gambling problem variable was aggregated for the community level variable.

The measure of gambling problems used in this study is not a measure of problem gambling prevalence, but is broadly consistent with the Australian national definition which states that *problem gambling is characterised by difficulties in limiting money and/or time spent on gambling which leads to adverse consequences for the gambler, others, or for the community*[27], p. 126. A more detailed discussion of the NLES, including its strength and weaknesses has been reported previously [10,12] and the NLES, as used in the HICH study, bas been found to be a reliable measure [25].

### **Explanatory variables**

Data on socio-demographic, socioeconomic, psychosocial, housing condition and community characteristics were collected. Child level variables used included age, sex, mobility, relationship to the carer, relationship to householder, breastfeeding history and day care attendance. Householder data was limited in its use in the analyses due to a number of variables having large amounts of missing data. Carer variables included age, sex, education, financial stress and psychosocial status.

Two psychosocial measures were collected for carers, in addition to the information collected as part of the NLES previously described. The Brief Screen for Depression (BSD) contains a set of questions scored on a Likert scale and when summed produce a maximum score of 50, with scores of 25 or greater indicating depression [28]. Positive and negative affect were measured using the two scales of the Affect Balance Scale [29], which gives scores of 0 to 4 (with 4 being most positive or most negative). Both scales were dichotomised with scores of 0 to 3 compared with scores of 4 (i.e. most negative or most positive). The BSD, Positive Affect Balance (PosAB), and Negative Affect Balance (NegAB) had Cronbach alpha scores (α = 0.57, α = 0.66, α = 0.58 respectively).

Data assessing both the hygienic state and physical functioning of various aspects of the house were collected according to two methods of rating houses that corresponded to twelve Healthy Living Practices (HLPs) as outlined in the National Indigenous Housing Guide [30], and an overall measure for the house. Briefly, the first method, known as the Failed HLP (FHLP), assessed the condition of individual items of infrastructure, which were then grouped according to the HLPs. Each HLP was then scored as a pass only if all items of infrastructure for the particular HLP passed. Eight out of the possible 13 HLPs were assessed using the FHLP method. The second method, known as the Surveyor Function Score (SFS), assessed the functional state of each HLP using a 7-point Likert scale that scored the infrastructure based on the perception of trained surveyors. A 7-point Likert scale was also used to assess the hygienic state of the house for each HLP and given by the Surveyor Condition Score (SCS). Full details of both HLP scoring methods are provided in more detail in previous publications [15,17,31].

Community level variables included in the analyses were: community ID (not identifiable), crowding, community housing function aggregated from eight of the HLPs, environmental health condition, whether the community has outstations (smaller communities on traditional homelands) attached, community location (desert, inland tropical, coastal tropical), frequency of access to a medical doctor, and community facilities (e.g. meeting hall, canteen (alcohol outlet), takeaway store, women’s centre and aged care facility).

### **Statistical analyses**

Two distinct analyses were undertaken to address the research questions. The first analysis used carer report of gambling problems for themselves or someone in their house as the outcome variable, which was a binary variable making it suitable for logistic regression modeling. Unadjusted associations were first calculated for community, carer, householder and dwelling level variables. Carer and householder variables showing moderately significant (p < 0.10) associations with reported gambling problems were then used to develop multivariable adjusted model for carer report of gambling problems. This was done by entering all moderately significant variables into the model and applying backward elimination with removal set at p > 0.05. Once the multivariable adjusted model for the carer report of gambling problems was determined, community level variables showing a significant bivariate association with reported gambling problems were entered separately into the model to determine if any community contexts retained significance after adjustment for carer and householder level characteristics. Plausible first order interactions were explored and pair-wise tests carried out if significant.

The second analysis was at the child level, with child illness (report of skin infection, scabies, respiratory infection, ear infection, diarrhoea and vomiting and two or more reports in the two weeks preceding the survey) used as the dependent variables’. Again, these outcome variables were binary and logistic regression modeling was used to assess unadjusted associations between child illness and community, carer, householder and dwelling level variables. The reported gambling problems variable was excluded from this part of the analyses, as this was a primary variable of interest and was added later to models. The following process was carried out for each of the child health outcomes. Carer, householder and dwelling level variables showing a moderately significant (p < 0.10) bivariate associations with child illness were retained for the next stage in the analysis. Because of the large number of variables, a blocked step approach was used to develop final multivariable adjusted models. First, demographic variables retained from the previous stage were entered simultaneously into a model and backward elimination carried out with removal set at p < 0.05. This procedure was then repeated separately for socioeconomic, psychosocial and dwelling variables. This procedure was also carried out for community level variables, though many variables exhibited co linearity and were excluded. Next, all significant variables from each of the domains were entered into a model simultaneously and backward elimination carried out with removal at p > 0.05. After arriving at this model, the carer and householder reported gambling problems were entered separately to determine if they retained a multivariable adjusted association with child health. Lastly, community level variables were added one at a time and included in final models if significant. Plausible first-order interactions were then tested and presented if significant.

Confidence intervals for all analyses were adjusted for clustering of children and carers in dwellings and communities using the Huber-White sandwich variance estimator. All analyses were carried out using Stata 9.2 IC.

## **Results**

Demographic data for carers and householders, and housing condition data have been presented in previously published papers (see [15,17,18]) and are only reported here if there was evidence of an effect in statistical models.

### **Reported gambling problems**

Table 1 shows the distribution of community level variables and unadjusted odds ratios (95% confidence intervals) for carer report of gambling problems. There was large variation in reported gambling problems, which ranged from 10% to 74%. Community level indicators showing evidence of increased odds for reported gambling problems included: poor community housing function, having the poorest environmental health, inland tropical communities, and not having a resident doctor. Community level indicators showing evidence of decreased odds in reporting gambling problems included: all communities, except communities 1 and 9, compared with community 3 (reference category); central Australian communities; communities with 1 to 5 outstations attached; and communities having a women’s centre and/or an aged care facility.

**Table 1:** Unadjusted logistic regression models for carer reported gambling problems and community level variables

**Independent variables**	**Carers**	**Carer gambling problems**^**1**^	**Unadjusted**
	**% (n)**	**% (n)**	**OR (95% CI)**^**2**^
**Total**	**347 (100.0)**	**40.4 (140)**	**-**
Community code			
1	14.2 (50)	52.0 (26)	0.38 (0.13–1.08)
2	9.9 (35)	45.7 (16)	**0.29 (0.10–0.86)**
3	9.9 (35)	74.3 (26)	1.0
4	5.7 (20)	30.0 (6)	**0.15 (0.04–0.52)**
5	12.8 (45)	44.4 (20)	**0.28 (0.10–0.77)**
6	3.1 (11)	18.2 (2)	**0.08 (0.01–0.44)**
7	14.5 (51)	10.0 (5)	**0.04 (0.01–0.13)**
8	17.1 (60)	37.9 (22)	**0.21 (0.07–0.60)**
9	7.7 (27)	48.2 (13)	0.32 (0.10–1.03)
10	5.1 (18)	25.0 (4)	**0.12 (0.03–0.45)**
Community FHLP^3^ measure			
Better (8 communities)	84.4 (297)	37.0 (108)	1.0
Worse (2 communities)	15.6 (55)	58.2 (32)	**2.37 (1.29–4.37)**
Community Environmental health			
Best (2 communities)	20.2 (71)	34.8 (24)	1.0
Better (4 communities)	37.2 (131)	29.7 (38)	0.79 (0.38–1.63)
Poor (2 communities)	18.5 (65)	40.0 (26)	1.25 (0.57–2.74)
Poorest (2 communities)	24.2 (85)	61.2 (52)	**2.95 (1.35–6.45)**
Number of outstations attached			
No outstations (2 communities)	35.8 (126)	49.2 (61)	1.0
1 to 5 (4 communities)	58.5 (206)	36.0 (73)	**0.58 (0.35–0.96)**
14 (2 communities)	5.7 (20)	30.0 (6)	0.44 (0.16–1.23)
Community location			
Desert (2 communities)	10.8 (38)	27.8 (10)	1.0
Inland-tropical (4 communities)	35.8 (126)	50.8 (64)	**2.68 (1.20–6.01)**
Coastal (4 communities)	53.4 (188)	35.7 (66)	1.44 (0.65–3.21)
Access to Doctor			
Resident (5 communities)	45.5 (160)	25.2 (39)	1.0
Twice or less/week (5 communities)	54.6 (192)	52.6 (101)	**3.30 (1.96–5.57)**
Community facilities present			
Women’s centre (7 communities)	76.7 (270)	35.9 (95)	**0.46 (0.27–0.79)**
Aged care (6 communities)	59.7 (210)	73.0 (73)	**0.59 (0.36–0.97)**

^1^ Carer reported gambling problems for themselves or someone in the house.

^2^ OR (95% CI) = odds ratio (95% confidence interval).

^3^*FHLP* Failed healthy living practices (housing infrastructure required to carry out health living practices). The FHLP measures the condition of housing infrastructure in the community [15].

Very few housing variables showed evidence of an association with reported gambling problems, so results are only presented in text. Poorer housing hygiene and infrastructure showed evidence of reduced odds in reports of gambling problems for the following housing variables: separate animals and humans SCS (OR 0.48, 95%CI 0.26 to 0.88), wash people FHLP (OR 0.55, 0.33 to 0.90), remove waste water SFS (OR 0.44, 0.26 to 0.73), control dust SFS (OR 0.56, 0.34 to 0.95) and house surrounds SFS (OR 0.58, 0.35 to 0.95). None of these variables remained in multivariable models after backward elimination (i.e. see Table 2).

**Table 2:** Multivariable logistic regression models for carer reported gambling problems

**Explanatory variables**	**Carers**	**Carer gambling problems**^**1**^	**Model 1 (n**_**carer**_ **= 336) Adjusted OR**	**Model 2 (n**_**carer**_ **= 336) Adjusted OR**
	**% (n)**	**% (n)**	**(95% CI)**^**1**^	**(95% CI)**^**2**^
Cohabitation with spouse				
No	25.1 (88)	30.2 (26)	1.0	ns
Yes	56.7 (199)	41.1 (81)	1.41 (0.77–2.59)	ns
Don’t know/Unsure	18.2 (64)	51.6 (33)	**3.25 (1.41–7.45)**	ns
Carer: # of children less than 7 years				
Less than three	48.7 (166)	31.9 (52)	1.0	1.0
Three or more children	51.3 (175)	46.8 (81)	**1.97 (1.15–3.37)**	**1.74 (1.00–3.02)**
Carer Negative Affect Balance				
Less negative	77.6 (263)	33.1 (86)	1.0	1.0
Most negative	22.4 (76)	65.8 (50)	**4.33 (2.45–7.67)**	**3.72 (2.08–6.66)**
Community access to doctor				
Resident	45.5 (160)	25.2 (39)	na	1.0
Twice or less per week	54.6 (192)	52.6 (101)	na	**3.10 (1.75–5.50)**
**Adjusted R**^**2**^			*9.6%*	*11.9%*

NOTE: *ns* Not significant (p > 0.05).

^1^ Carer reported gambling problems for themselves or someone in the house.

^2^*OR* Odds Ratio (95% confidence interval).

Table 2 presents two multivariable adjusted logistic regression models for carer report of gambling problems. Model 1 includes carer level variables only, and had an adjusted R^2^ of 9.6%. No housing variables remained in final multivariable models. Model 2 included three variables showing evidence of an association with reported gambling problems. The largest effect size was for carer’s who were most negative on the NegAB Scale (OR 4.33, 2.45 to 7.67), followed by carer’s not knowing/unsure whether their spouse was cohabiting with them (OR 3.25, 1.41 to 7.45), and lastly carer’s with three or more children (OR 1.97, 1.15 to 3.37). Model 2 shows the multivariable model after inclusion of significant community level variables and has an adjusted R^2^ of 11.9%. There was evidence of communities with less access to a doctor reporting more gambling problems (OR 3.10, 1.75 to 5.50), and the inclusion of this variable reduced the effect size for carer NegAB and carer number of children, while carer cohabitation with spouse became non-significant and was dropped. There were no evidence of interactions in these models.

### **Child illness**

Tables 3 and 4 show unadjusted logistic regression models for each child health outcome and carer, householder and community level reported gambling problems. There was evidence for carer report of gambling problems being positively associated with two or more reports of illness (OR 1.77, 1.21 o 2.60), skin infections (OR 1.86, 1.17 to 2.95), and ear infections (OR 1.50, 1.00 to 2.26). There was evidence of a positive association between householder reported gambling problems and two or more reports of illness (OR 1.75, 1.18 to 2.60), scabies (OR 1.94, 1.17 to 3.20), diarrhoea and/or vomiting (OR 1.47, 1.00 to 2.18), and ear infections (OR 1.72, 1.16 to 2.57). For community level gambling problems there was evidence of a positive association with two or more reports of illness (OR 2.01, 1.09 to 3.69), scabies (OR 2.07, 1.21 to 3.53) and ear infections (OR 2.29, 1.26 to 4.15).

**Table 3:** **Unadjusted logistic regression models for carer report of child health (dependent variable) and carer, householder and community reported gambling problems (n**_**children**_ **= 618)**

	**2 or more reports**	**Skin infections**	**Scabies**^**2**^
	**OR (95% CI)**	**OR (95% CI)**	**OR (95% CI)**
Carer gambling problems^3^	**1.77 (1.21–2.60)**	**1.86 (1.17–2.95)**	1.18 (0.74–1.87)
Householder gambling problems^4^	**1.75 (1.18–2.60)**	1.11 (0.69–1.81)	**1.94 (1.17–3.20)**
Community level gambling problems			
Lowest problems (<20%)	(p = 0.08) 1.0	(p = 0.30) 1.0	1.21 (0.60–2.40)
Intermediate problems (20% to 49%)	1.39 (0.83–2.33)	0.65 (0.35–1.21)	(p = 0.03) 1.0
Highest problems (>50%)	**2.01 (1.09–3.69)**	0.91 (0.45–1.83)	**2.07 (1.21–3.53)**

^1^ Skin infections with no scabies.

^2^ Scabies with or without skin infections.

^3^ Carer reported gambling problems for themselves or someone in the house.

^4^ Householder reported gambling problems for themselves or someone in the house.

**Table 4:** **Unadjusted logistic regression models for carer report of child health (dependent variable) and carer and householder report of gambling problems (n**_**children**_ **= 618)**

	**Diarrhoea &/or vomiting**	**Respiratory infections**	**Ear infections**
	**OR (95% CI)**	**OR (95% CI)**	**OR (95% CI)**
Carer gambling problems^1^	1.43 (0.96–2.12)	1.37 (0.92–2.05)	**1.50 (1.00–2.26)**
Householder gambling problems^2^	**1.47 (1.00–2.18)**	1.12 (0.74–1.69)	**1.72 (1.16–2.57)**
Community level gambling problems			
Lowest problems (<20%)	(p = 0.97) 1.0	(p = 0.14) 1.0	(p = 0.01) 1.0
Intermediate problems (20% to 49%)	1.01 (0.61–1.69)	1.66 (0.94–2.92)	1.28 (0.75–2.18)
Highest problems (>50%)	1.07 (0.59–1.94)	1.85 (0.97–3.55)	**2.29 (1.26–4.15)**

^1^ Carer reported gambling problems for themselves or someone in the house.

^2^ Householder reported gambling problems for themselves or someone in the house.

Tables 5 and 6 present the multivariable adjusted models for scabies and ear infection. Two multivariable models are reported for scabies in Table 5. Model 1 does not include the gambling problems variable and had an adjusted R^2^ of 6.1%, while model 2 includes householder reported gambling problems and has an adjusted R^2^ of 8.3%. After controlling for other predictors of scabies, there was evidence that householder reported gambling problems having a positive association with carer report of scabies in children (OR 1.81, 1.07 to 3.05). Only one housing variables retained a significant multivariable association with scabies in children and was the separate humans and animals SCS (OR 0.49, 0.28 to 0.86). Other variables remaining in this model were carer cohabitation with spouse, and child relationship to the householder. No community level variables remained in this model after adjustment for other variables and there was no evidence for interactions.

**Table 5:** Multivariable adjusted logistic regression models for carer report of scabies in the child over the 2 weeks prior to the survey

			**Model 1**	**Model 2**
	**Children**	**Scabies**	**Scabies (n = 577)**	**Scabies (n = 565)**
**Explanatory variables**	**n (%)**	**n (%)**	**OR (95% CI)**	**OR (95% CI)**
Householder reported gambling problems				
No gambling problems	299 (52.9)	38 (12.7)	na	1.0
Yes - gambling problems	266 (47.1)	57 (21.4)	na	**1.81 (1.07–3.05)**
Carer cohabits with spouse				
No	126 (22.3)	28 (22.2)	1.0	**1.0**
Yes	337 (59.7)	39 (11.6)	**0.53 (0.31–0.93)**	**0.43 (0.24–0.77)**
Don’t know	102 (18.1)	28 (27.5)	1.11 (0.53–2.29)	0.98 (0.47–2.06)
Child relationship to householder				
Son/daughter	160 (28.3)	17 (10.6)	1.0	1.0
Grandson/Grand-daughter	291 (51.5)	61 (21.0)	**2.06 (1.12–3.79)**	**1.89 (1.00–3.55)**
Nephew/niece	87 (15.4)	16 (18.4)	1.63 (0.75–3.53)	1.71 (0.78–3.76)
Other	27 (4.8)	1 (3.7)	0.51 (0.11–2.41)	0.24 (0.03–2.00)
Separate animals & humans hygiene				
Scores 1–3 (better)	115 (20.4)	33 (28.7)	1.0	1.0
Scores 4–7 (worse)	450 (79.7)	62 (13.8)	**0.47 (0.27–0.83)**	**0.49 (0.28–0.86)**
**Total/Adjusted R**^**2**^	**565 (100.0)**	**95 (16.8)**	*6.1%*	*8.3%*

NOTE: *na* Not applicable; *ns* Not significant (p>0.05).

**Table 6:** **Multivariate adjusted logistic regression models for carer report of** ***ear infections*** **for the child over the previous 2 weeks to the survey**

			**Model 1**	**Model 2**	**Model 3**
	**Children**	**Ear infections**	**Ear infections**	**Ear infections**	**Ear infections**
			**(n = 556)**	**(n = 539)**	**(n = 556)**
**Explanatory variables**	**n (%)**	**n (%)**	**OR (95% CI)**	**OR (95% CI)**	**OR (95% CI)**
Community level gambling problems					
Lowest problems (<20%)	96 (17.3)	21.9 (21)	na	na	1.0
Intermediate (20–49%)	335 (60.3)	27.2 (91)	na	na	1.20 (0.65–2.20)
Highest problems (>50%)	125 (22.5)	40.0 (50)	na	na	**2.24 (1.18–4.26)**
HH reported gambling problems					
No gambling problems	285 (52.9)	68 (23.9)	na	1.0	ns
Yes - gambling problems	254 (47.1)	91 (35.8)	na	**1.68 (1.09–2.58)**	ns
Child age (years)					
Less than 1 year	79 (14.7)	14 (17.7)	1.0	1.0	1.0
1 - 2 years	169 (31.4)	69 (40.8)	**2.84 (1.52–5.31)**	**2.70 (1.44–5.08)**	**3.05 (1.63–5.72)**
3 - 7 years	291 (54.0)	76 (26.1)	1.44 (0.77–2.69)	1.43 (0.76–2.68)	1.56 (0.84–2.90)
Child in day care					
Not in day care	487 (90.4)	132 (27.1)	1.0	1.0	1.0
At least one day per week	52 (9.7)	27 (51.9)	**2.86 (1.46–5.60)**	**2.85 (1.41–5.76)**	**2.60 (1.32–5.14)**
Carer relationship to householder					
Same	142 (26.4)	36 (25.4)	1.0	1.0	1.0
Daughter	139 (25.8)	48 (34.5)	1.22 (0.68–2.18)	1.23 (0.68–2.22)	1.11 (0.62–1.98)
Wife	108 (20.0)	31 (28.7)	0.93 (0.47–1.83)	1.05 (0.51–2.15)	0.92 (0.46–1.82)
Daughter-in-law	27 (5.0)	6 (22.2)	0.63 (0.22–1.77)	0.65 (0.21–1.96)	0.61 (0.21–1.75)
Niece	33 (6.1)	4 (12.1)	**0.32 (0.12–0.86)**	**0.29 (0.11–0.76)**	**0.27 (0.11–0.70)**
Sister	21 (3.9)	12 (57.1)	2.95 (0.95–9.13)	2.99 (0.92–9.71)	2.88 (0.93–8.88)
Sister-in-law	13 (2.4)	4 (30.8)	1.22 (0.30–5.03)	1.26 (0.29–5.52)	1.14 (0.28–4.73)
Other	56 (10.4)	18 (32.1)	1.19 (0.56–2.51)	1.13 (0.51–2.50)	1.12 (0.54–2.35)
Failed healthy living practices					
0 to 3	90 (16.7)	17 (18.9)	1.0	1.0	1.0
4 to 8	449 (83.3)	142 (31.6)	**2.02 (1.19–3.44)**	**2.20 (1.26–3.83)**	**1.99 (1.15–3.43)**
**Total/Adjusted R**^**2**^	**539 (100.0)**	**159 (29.5)**	*7.2%*	*8.6%*	*8.5%*

NOTE: *na* not applicable; *ns* not significant (p > 0.05).

Table 6 presents three multivariable models for carer report of ear infections in children. Model 1 excludes reported gambling problems (and community variables) and had an adjusted R^2^ of 7.2%. When added to this model (model 2), there was evidence that householder reported gambling problems was positively associated with ear infections (OR 1.68, 1.09 to 2.58). The overall house FHLP variable showed evidence of association with ear infections, with children in poorer functioning houses having increased odds of a carer report of an ear infection (OR 2.20, 1.26 to 3.83). Other variables in the model showing evidence of an association with ear infections were child age, day care centre attendance and carer relationship to the householder. Model 2 had an adjusted R^2^ of 8.6%. The community level gambling problems was added to this model, which led to the householder report of gambling problems becoming non-significant and being dropped (model 3). There was evidence that children living in communities where more than 50% of carers reported gambling problems had increased odds of ear infection (OR 2.24, 1.18 to 4.26). All other variables from model 2 remained in model 3, which had an adjusted R^2^ of 8.5%. There was no evidence of any other community level variables being associated with ear infections and there was no evidence for interactions.

## **Discussion**

### **Child health and gambling problems in households**

The empirical analyses support the anecdotal evidence from the *Little Children are Sacred* report and the more recent review of child protection services in the NT [23,24] that gambling is associated with child health. Specifically, household gambling problems were positively associated (after adjustment for other significant predictors) with carer report of ear infections and scabies in children. The relationship between gambling problems and infectious disease could be a result of children being exposed to other gamblers who may be carriers. For example, if the house is a regular card gambling venue there would be a steady flow of visitors through the house who would be using household facilities such as the toilet, linen and towels etc. and if contagious, greatly increase the chances of transmission to children in the house [20,21,32]. This could explain the lack of a multivariable adjusted association with the measures of crowding used (i.e. number of adults in house, number of children less than 7 years and persons per bedroom) and child health outcomes. That is, it may not be crowding per se, but the transient flow of people associated with gambling in the house causing transmission of infections. However, it may also be that gambling problems are more likely to occur in overcrowded houses, with problems affecting carer’s of children, regardless of their own gambling practices, all contributing to poor child health and neglect [6].

The problems that carers’ were referring to in the gambling problems variable are unknown, but are likely to be similar to those identified in previous research [4,6-8,33]. They include physical and emotional child neglect, running short of money for essentials, loud card games going for days affecting children’s sleep and school attendance, demand sharing, increased community and family stress, exploitation of disadvantaged members in the community, domestic violence, large winnings from gambling being taken out of the community to be spent on alcohol, and neglect of houses. The cycle of dependency often associated with Indigenous gambling in remote communities has been well identified [3,4,8,34], and it is most likely that these cycles, along with the factors just outlined create an environment conducive to transmission of infections in children living in communities experiencing these problems [17,20,22].

Two variables in the model for carer report of scabies showed evidence of providing protection for children having a report of scabies by their carer. Children had reduced odds of a carer report of scabies if their carer cohabited with their spouse, and if they lived in a house that had poor control of hygiene in separating animals and humans. Though speculative, possible reasons why cohabiting with a spouse is protective for these children include the ability of a male head to exert some degree of control about what happens in the house and increased available income. The association with hygiene control over separating humans and animals is surprising, but may relate to the location of the community and the types of housing provided. Certainly, there was variation across communities in a range of housing and community variables [15,18], and a more focused community level analyses would be required to tease out these relationships.

### **Gambling problems, community contexts and carer characteristics**

The significant variation between communities for carer report of gambling problems in households ranged from 10% to 74% and highlights the diversity between remote communities in the NT. A large number of the community level variables showed evidence of an unadjusted association with reported gambling problems, though only community access to a medical doctor remained in multivariable models for reported gambling problems. The multivariable model for carer reported gambling problems contained community access to doctor, carer cohabitation with spouse unknown, three or more young children in the house (a measure of crowding), and poor carer psychosocial health. These associations reinforce that gambling, as an activity, does not occur in isolation of the social and physical aspects of the community, which can influence both propensity for people to gamble and consequences (harmful or not) resulting from excessive gambling [3,5,10,11,34]. An analysis of the National Aboriginal and Torres Strait Islander Social Survey at both the national and NT level also found that community level variables, namely having gang, family violence, alcohol and physical assault problems in the community showed evidence for a multivariate adjusted association with increased reporting of gambling problems [10,11]. Unfortunately, the HICH data set did not contain community level variables on specific community problems, so these associations were unable to be tested in the current analyses.

There are a number of possible explanations for the finding that communities with a resident doctor had less than half the reported gambling problems as those where the doctor visited twice or less a week (53% cf. 25%). First, it may be that these communities are more functional on a general level and are able to maintain the services of a full-time doctor, which is a significant problem in remote communities [35]. Second, these communities may be running concurrent services that provide general public health messages, with residents in these communities having more awareness about harms associated with gambling. No definitive answer can be given and more detailed studies are required to further understand this finding.

Some 18% of carers reported that they were unsure of their living arrangements with their spouse; with 52% of these reporting gambling problems. There could be a number of reasons for this finding and it is not possible to be definitive on the reasons for why carers’ were unsure of their living arrangements, as no further information was collected in this study. However, it may indicate that the carer is in an unstable relationship with their spouse, and the possibility of domestic violence [23] may be affecting their ability to care for their children. In analyses of a national representative survey, Stevens and Young [10,12] found that people reporting gambling problems for themselves, a family member or a close friend were more likely to report alcohol and drug problems, trouble with the police, being a witness to violence, and being abused or in a violent crime. The clustering of items relating to *social transgressions* was consistent for the Australian remote and non-remote Indigenous populations and for the NT Indigenous population. Furthermore, gambling problems were reported at higher levels in this study for carer’s with poor NegAB. People with gambling problems often also report a range of co-morbidities including anxiety and depression, tobacco use, and alcohol and drug dependence [36-38], with the findings herein consistent with this often observed finding.

As the analyses in this study were cross-sectional, it is not possible to know the direction of causation between gambling problems and poor carer NegAB, and it may be that gambling problems precede poor psychosocial health. An analysis currently in progress using the same data set is focusing on carer’s psychosocial health status as the outcome, which will give a better understanding of the relationship between gambling problems and psychosocial health.

One measure of crowding, number of children less than 7 years in the house, showed evidence of a multivariable adjusted association with carer reported gambling problems. This can be interpreted two ways. First, it may indicate that carers with more children are more concerned about the impacts of gambling and are reporting at higher levels. On the other hand, these carers are experiencing more problems related with the gambling itself, due to the negative consequences of problem gambling, such as shortages of money, increased domestic violence and neglect of children [3,4]. The paucity of good quality comprehensive studies on gambling within Indigenous communities in the NT highlights the difficulty of doing research on this topic in these communities. Indigenous people in these communities are often reluctant to talk about gambling, which may be a product of the people themselves, identifying with the card games as being distinctly Indigenous, and hence, not the business of a non-Indigenous researcher. Indeed, having strong ties and good relations over a significant period of time are a requirement of carrying out successful research in Indigenous communities [39].

The evidence for the complex associations between gambling problems, child health and community contexts point to a need for a comprehensive strategy to reduce the harm associated with gambling in remote communities. This would best be served by application of a holistic public health model incorporating prevention, treatment, regulation and education [40,41].

### **Educational approaches to gambling harm minimisation**

There is a clear need for improved levels of understanding of the negative consequences arising from over participation in gambling amongst Indigenous people living in remote communities. This includes community card games and commercial gambling, as it is likely that both forms of gambling are leading to harm. There is a need for communities to be more open in discussing gambling and the negative consequences of over-participation, along with strategies to mitigate harm. To encourage discussion of gambling and related problems, it is necessary to ensure people understand the negative consequences of gambling. The findings of this report go some way in identifying some of the negative consequences of excessive gambling on children. The inter-generational normalisation of gambling and acceptance of associated harms has been exacerbated through early exposure to gambling in remote communities [3,34] would suggest that education programs also target youth. Currently, Amity Community Services (located in Darwin) run gambling-related programs in Indigenous communities in the NT. These programs involve training health workers to identify problem gamblers, and running education on concepts associated with gambling and the negative consequences associated with excessive gambling (*personal communication*, Kylie Jericho, Amity Community Services).

### **Social regulation in remote communities as a strategy for reducing gambling harm**

Research in north Queensland communities found normative or social regulation of card games [3,33] quite common. Using these existing structures, such normative approaches could be further refined to widen the impact of social regulation. Table 7 summarises social regulation as identified in previous research, and outlines areas that could be discussed with community residents and health workers to increase the reach of this harm reduction strategy. These social regulation strategies provide for harm reduction and are consistent with public health approaches to minimising harm associated with gambling [3,40].

**Table 7:** Normative regulation and gaps in normative regulation in community gambling

**Existing social regulation**	**Additional social regulation for consideration**
No drinking or drunk players	Days/times of play for gambling
No arguing	Locations for gambling (fixed places)
High versus low stake games	Limiting stakes, particularly high stake games
Family/kin verse general open games^1^	Age restrictions
Collecting a tong^2^	Adequate child minding facilities

^1^ Family/kin games are usually lower stake games and are a sociable activity, while general games more likely to be high stakes games where elements of kinship responsibilities are not adhered to [4,8,9,34].

^2^ A tong is a small fee card players pay to enter the card game (usually held in a house), and players may receive cups of tea and biscuits.

Using the finding that gambling problems are related to poor child health and poor psychosocial health in carers could be a starting point for discussion with community residents and health workers in drawing attention to gambling related problems. This information, along with a discussion on the merits of the additional social regulation proposed above will give communities some ammunition in addressing gambling problems. While the list of additional social regulations may not be palatable to many gamblers in remote communities, there is a need for people to be informed about the negative consequences arising from excessive gambling and to formulate ways in which negative consequences can be minimised.

### **Limitations of the research**

There are three caveats to the analyses that limit the strength of the evidence for gambling-related problems in households being a risk factor for poor child health. First, the question on gambling problems used in the survey is subjective and there would be variation in what constitutes a problem between communities and people within communities. In the current study the percentage of carers reporting gambling problems for themselves or someone in their household ranged from 10% to 74%, which would indicate that gambling problems are significant issues for the Aboriginal people in the study, at least in some communities. Further, the NLES module in which the gambling problems variable is an item, has been shown to be a reliable measure for the current data set [26], and is consistent with results using the same module in national surveys of the Indigenous population [10,12].

Second, the type of gambling activity causing the problems is unknown and could be either commercial venue gambling or community card games. Of the ten communities included in the study, eight were more than 100 km from the nearest regional centre (average distance = 240 km), so access to commercial forms of gambling were limited, implying that problems are more likely related to community card games. Though further studies are required to assess definitively, what type of gambling activities are the predominant causes of problems, but it is most likely that both commercial and non-commercial forms of gambling are causing problems. Third, the analyses were cross-sectional, so causal relationships were unable to be assessed. However, the analyses did show that gambling problems were independently associated with poor child health and analyses are currently underway utilising the longitudinal nature of the data set.

## **Conclusions**

This research identifies statistically significant associations between poor child health outcomes and reported gambling problems in households in remote Indigenous communities, after adjustment for other covariates of poor child health. The high levels of reported gambling problems in remote communities and the associations observed between gambling problems, community contexts, carer psychosocial health and child health all lead to the conclusion that gambling is causing significant harm. There is a need to implement comprehensive public health approaches to harm minimisation associated with gambling in remote Indigenous communities, and these approaches need to link closely with other measures to improve community safety.

## **Abbreviations**

ABS, Australian Bureau of Statistics; FHLP, Failed Healthy Living Practice; HICH, Housing Improvement and Child Health in Aboriginal communities study; HLP, Healthy Living Practice; NegAB, Negative Affect Balance; NLES, Negative Life Events Scale; NT, Northern Territory; OR, Odds Ratio; PosAB, Positive Affect Balance; SCS, Surveyor Condition Score; SFS, Surveyor Function Score.

## **Competing interests**

The authors declare that they have no competing interests.

## **Authors’ contributions**

MS completed all data analyses, interpretation of results and full drafting of the manuscript. RB conceptualised and designed the HICH study. All authors read and approved the final manuscript.

## Pre-publication history

The pre-publication history for this paper can be accessed here:

http://www.biomedcentral.com/1471-2458/12/377/prepub
